# Chromosome level genome assembly of the Etruscan shrew *Suncus etruscus*

**DOI:** 10.1038/s41597-024-03011-x

**Published:** 2024-02-07

**Authors:** Yury V. Bukhman, Susanne Meyer, Li-Fang Chu, Linelle Abueg, Jessica Antosiewicz-Bourget, Jennifer Balacco, Michael Brecht, Erica Dinatale, Olivier Fedrigo, Giulio Formenti, Arkarachai Fungtammasan, Swagarika Jaharlal Giri, Michael Hiller, Kerstin Howe, Daisuke Kihara, Daniel Mamott, Jacquelyn Mountcastle, Sarah Pelan, Keon Rabbani, Ying Sims, Alan Tracey, Jonathan M. D. Wood, Erich D. Jarvis, James A. Thomson, Mark J. P. Chaisson, Ron Stewart

**Affiliations:** 1 Regenerative Biology, Morgridge Institute for Research, 330 N. Orchard St., Madison, WI 53715 USA; 2 Neuroscience Research Institute, University of California - Santa Barbara, 494 UCEN Rd, Isla Vista, CA 93117 USA; 3 Department of Comparative Biology and Experimental Medicine, University of Calgary, 2500 University Drive NW, Calgary, Alberta T2N 1N4 Canada; 4 Vertebrate Genome Lab, The Rockefeller University, 1230 York Avenue, New York, NY 10065 USA; 5 BCCN/Humboldt University Berlin, Philippstr, 13 House 6, 10115 Berlin, Germany; 6 Max Planck Institute for Biology Tübingen, Max-Planck-Ring 5, 72076 Tübingen, Germany; 7 Laboratory of Neurogenetics of Language, The Rockefeller University/HHMI, 1230 York Avenue, New York, NY 10065 USA; 8 DNAnexus Inc., 1975 W El Camino Real, Mountain View, CA 94040 USA; 9 Department of Computer Science, Purdue University, 249 S. Martin Jischke Dr, West Lafayette, IN 47907 USA; 10 LOEWE Centre for Translational Biodiversity Genomics, Senckenberganlage 25, 60325 Frankfurt, Germany; 11 Senckenberg Research Institute, Senckenberganlage 25, 60325 Frankfurt, Germany; 12 Institute of Cell Biology and Neuroscience, Faculty of Biosciences, Goethe University Frankfurt, Max-von-Laue-Str. 9, 60438 Frankfurt, Germany; 13 Tree of Life, Wellcome Sanger Institute, Cambridge, CB10 1SA UK; 14 Department of Biological Sciences, Purdue University, 249 S. Martin Jischke Dr., West Lafayette, IN 47907 USA; 15 Department of Quantitative and Computational Biology, University of Southern California, 1050 Childs Way RRI 408, Los Angeles, CA 90089 USA; 16 Department of Molecular, Cellular and Developmental Biology, University of California Santa Barbara, Santa Barbara, CA 93106 USA; 17 Department of Cell and Regenerative Biology, University of Wisconsin School of Medicine and Public Health, Madison, WI 53726 USA

**Keywords:** Genome, DNA sequencing

## Abstract

*Suncus etruscus* is one of the world’s smallest mammals, with an average body mass of about 2 grams. The Etruscan shrew’s small body is accompanied by a very high energy demand and numerous metabolic adaptations. Here we report a chromosome-level genome assembly using PacBio long read sequencing, 10X Genomics linked short reads, optical mapping, and Hi-C linked reads. The assembly is partially phased, with the 2.472 Gbp primary pseudohaplotype and 1.515 Gbp alternate. We manually curated the primary assembly and identified 22 chromosomes, including X and Y sex chromosomes. The NCBI genome annotation pipeline identified 39,091 genes, 19,819 of them protein-coding. We also identified segmental duplications, inferred GO term annotations, and computed orthologs of human and mouse genes. This reference-quality genome will be an important resource for research on mammalian development, metabolism, and body size control.

## Background & Summary

The Etruscan shrew (*Suncus etruscus*), also known as the white-toothed pygmy shrew, is recognized as one of the smallest mammals living today. With a body weight ranging from 1.2 to 2.7 grams and dimensions spanning 36 to 53 mm in length^1^, this organism exhibits a remarkably large body surface area to volume ratio. As a result, the shrew has an exceptionally high basal metabolic rate, which requires a daily food consumption approximating 1.5 to 2.0 times its body mass^1^. Due to these unique physiological characteristics, the Etruscan shrew has become a valuable species to the scientific community, significantly contributing to various fields of research, such as behavioral science and neuroscience^1–4^. A high-quality genome assembly is an essential reference to enable accurate high throughput data analysis. It will provide valuable insights into the mechanisms of body size control and metabolic rate, as well as facilitate comparative biological investigations.

Our new *Suncus etruscus* genome is the first chromosome-level genome assembly of the order *Eulipotyphla*. *S. etruscus* is a member of the family *Soricidae* (the shrews), which have classically been divided into subfamilies *Crocidurinae* (the white-toothed shrews) and *Soricinae* (the red-toothed shrews). An alternative partitioning scheme distinguishes three subfamilies of the *Soricidae*, namely *Crocidurinae* (the white-toothed shrews), *Soricinae* (the red-toothed shrews) and *Myosoricinae* (African shrews)^5^. *S. etruscus* is a member of the *Crocidurinae*, which total about 220 species, representing a substantial portion of mammalian diversity. At the time of writing, there were several other sequenced shrew genomes: *Crocidura indochinensis*^6^, *Cryptotis parvus*^7,8^, *Sorex araneus*^9,10^, *Sorex fumeus*^11,12^, and *Sorex palustris*^13^. As discussed in the Technical Validation section, these genome assemblies were based on Illumina short read data, sometimes in combination with long-range technologies such as Nanopore long reads or Hi-C, which enabled scaffolding but fell short of chromosome-level assembly. *C. parvus* is also a very small species – which makes it an interesting comparison with *S. etruscus*. *C. parvus* is a member of the subfamily *Soricinae* (the red-toothed shrews). The *Soricinae* are generally thought to have a higher metabolism than *Crocidurinae*. It is clear, however, that *Suncus etruscus* – as a collateral of its small size – has a particularly high metabolic rate and also shows neural specializations for metabolic control^14^.

We sequenced and assembled the Etruscan shrew genome, of a male, using protocols developed by the Vertebrate Genomes Project (VGP) to generate a reference-quality genome assembly^15^. Briefly, we used a combination of PacBio Continuous Long Read (CLR) sequencing, 10X Genomics linked reads, Bionano Genomics optical maps, and Arima Genomics Hi-C linked reads. PacBio reads were used to build the contigs and generate a pseudo-haplotype assembly, with a 2.472 Gbp primary and 1.515 Gbp alternate. 10x linked reads, optical maps, and Hi-C were used for scaffolding, and 10x linked reads were used to simultaneously polish the primary and the alternate assemblies. The primary assembly was manually curated, correcting 212 missing or missed joins, removing 28 sequences representing false haplotypic duplication, and assigning 99.9% of the sequence to 22 chromosomes, including X and Y. This karyotype was consistent with prior cytological studies^16–18^. The resulting reference assembly was highly contiguous, with scaffold N50 of 132 Mbp and contig N50 of 5 Mbp. Upon deposition to NCBI, it was annotated by the NCBI Eukaryotic Genome Annotation and Ensembl Rapid Release pipelines. The NCBI annotation pipeline identified 39,091 genes, 19,819 of them protein-coding. Ensembl Genebuild identified 37,534 genes, 19,562 protein-coding genes, 17,147 non-coding genes, and 825 pseudogenes.

We next computationally inferred Gene Ontology (GO) terms for the protein-coding genes predicted by NCBI using software developed in the Kihara Lab, including Protein Function Prediction (PFP)^19^, Phylogenetic tree-based Protein Function Prediction (Phylo-PFP)^20^, and Extended Similarity Group (ESG)^21^. Consensus GO terms were assigned to 26,579 protein products. We also computed protein-coding gene annotations and human/mouse orthologs using the Tool to infer Orthologs from Genome Alignments (TOGA)^22^. We used TOGA annotations of a set of ancestral mammalian genes to compare the quality of our assembly to other *Eulipotyphla* genomes, as discussed in the Technical Validation section below. Finally, we annotated segmental duplications as previously described^23,24^. Briefly, we identified resolved duplications by a whole genome self-alignment and collapsed ones by mapping CLR reads to the assembly and determining read depth using a hidden Markov model. We then used NCBI RefSeq annotations to identify genes that mapped to duplicated segments of the genome. Etruscan shrew has significantly fewer duplicated genes compared to several previously annotated species of rodents and artiodactyls^23,24^. GO terms, TOGA, and segmental duplications add significant value to the standard annotations provided by NCBI and Ensembl.

## Methods

### Sample collection and ethics statement

One adult male Etruscan shrew (*Suncus etruscus*) was provided by Dr. Michael Brecht, Bernstein Center for Computational Neuroscience, Humboldt University, Berlin, Germany. The shrew was captive-born and housed in Dr. Brecht’s colony^25^. All procedures complied with German regulations on animal welfare and were approved by an ethics committee^26^. Etruscan shrew tissue was collected according to a permit T0078/16 given to the Brecht group.

The Etruscan shrew was euthanized using an overdose of isoflurane and dissected under a microscope. Skin, heart, lung, and muscle tissue were collected for primary fibroblast culture, which would provide an unlimited source of cellular material for genomic and developmental studies. The shrew tissues were transferred into separate tubes containing ice-cold Alpha-MEM (Corning) with 1x Antibiotic-Antimitotic (Life Technologies). Tissues were minced individually with a scalpel and digested for 30 minutes at 37 °C in 0.5 ml of a 0.125 mg/ml solution of Liberase TM (Roche). 5 ml of pre-warmed fibroblast medium composed of a 50:50 mix of Alpha-MEM (Corning), 10% fetal bovine serum (Millipore) with 1x Antibiotic-Antimitotic (Life Technologies) and FBM complete (LONZA) was added to each digested tissue sample and transferred to gelatin-coated T25 tissue culture flasks (Corning). Spent medium was replaced carefully every other day without disturbing the adhering tissue pieces. After 7 days of incubation and maintenance at 37 °C, 5% CO_2_, 4% O_2_, a lung fibroblast culture began to develop. The remaining tissues failed to yield cell cultures and were discarded. Once the lung fibroblast culture reached confluency, it was passaged, banked, expanded, and sent to the Rockefeller University for genomic DNA isolation.

DNA isolation was performed at the Rockefeller University Vertebrate Genome Lab. Two million cells stored at −80 °C were used to extract high molecular weight DNA with the Bionano SP Blood and Cell Culture DNA Isolation Kit (Bionano PN 80042) following manufacturer’s protocols. This method utilizes gentle lysis and Nanobind magnetic disks to prevent DNA breakage and preserve large fragment lengths (>100–300 kb) needed for long-read sequencing.

### Genome sequencing and assembly

**Fig. 1:**
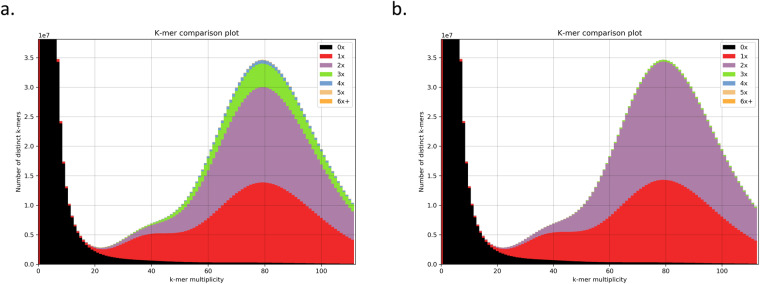
Removal of false duplications confirmed by k-mer spectra. K-mer spectra before (**a**) and after (**b**) false duplication removal.

PacBio and 10X sequencing, optical mapping, and Hi-C generation were performed by the Rockefeller University Vertebrate Genome Laboratory using standard VGP protocols as previously described in Secomandi *et al*.^27^. The genome was assembled as previously described in Secomandi *et al*.^27^, with minor modifications. Prior to the assembly, Genomescope2.0^28^ was used on the raw 10X reads, yielding, through statistical analyses of *k*-mer profiles, an estimated genome size of 2.65 Gbp, heterozygosity of 0.22%, and repeat content of 0.75 Gbp. Genomescope2.0 was run with K = 31 on the histogram generated with Meryl version 1.0.0^29^ using the 10X linked reads with barcodes (i.e., the first 23 bp of the forward read) trimmed off. Full details are available on VGP GenomeArk (https://genomeark.s3.amazonaws.com/index.html?prefix=species/Suncus_etruscus/mSunEtr1/assembly_vgp_standard_1.7/evaluation/genomescope/union_meryl_gs/). The assembly was performed on the DNAnexus cloud-based informatics platform for genomic data analyses (https://www.dnanexus.com) using the VGP standard genome assembly pipeline version 1.7 (https://github.com/VGP/vgp-assembly)^15^. PacBio subreads were used in the first FALCON version 2.0.2^30^ contigging step. Pre-assembled contigs underwent a phasing step with FALCON-unzip version 8.0.1^31^ (smrtanalysis v3.0.0) and a first round of Arrow^30^ (smrtanalysis version 5.1.0.26412) polishing. FALCON version 2.0.2 and FALCON-unzip version 8.0.1 were run with default parameters, with the exception of parameters related to the identification of read overlaps, which were adjusted as described in Secomandi *et al*.^27^. FALCON-unzip generated a set of primary contigs representing the primary pseudo-haplotype, and a set of alternate haplotigs, representing the secondary haplotypes. Purge_dups version 1.0.0^32^ was run to identify and remove false duplications. This was confirmed by the removal of most 3- and 4-copy *k*-mers, as evidenced by *k*-mer spectra computed and visualized using KAT version 2.4.2^33^ (Fig. 1).

After removing false duplications, a three-steps scaffolding strategy was performed on the purged primary contigs using Illumina short-reads (10x Genomics), Bionano optical maps and Hi-C reads. Two scaffolding rounds with scaff10X version 2.0.3 (https://github.com/wtsi-hpag/Scaff10X) were performed with options -matrix 2000 -reads 12 -link 10 and then -matrix 2000 -reads 8 -link 10. The resulting intermediate was then scaffolded with Bionano DLS optical maps^34^ using Bionano Solve version 3.4.0 in non-haplotype assembly mode with a DLE-1 one enzyme non-nicking approach. Finally, Hi-C scaffolding was performed as described in Secomandi *et al*.^27^. Briefly, Hi-C reads from Arima were aligned with the Arima Genomics mapping pipeline (https://github.com/ArimaGenomics/mapping_pipeline) and scaffolded with Salsa version 2.2^35^ with -m yes -i 5 -p yes parameters and -e GATC,GANTC,CTNAG,TTAA as restriction enzymes. In order to improve per-base accuracy (QV)^15^, the assembly was polished as previously described in Secomandi *et al*.^27^. To prevent haplotype switches and overpolishing of nuclear mitochondrial DNA segments (NUMTs)^15,36^, the scaffolded primary assembly was merged with alternate combined haplotigs. The combined intermediate was polished with gcpp version 2.0.2 (pacific Biosciences; smrtanalysis version 5.1.0.26412) with the command ‘pbalign --minAccuracy = 0.75 --minLength = 50 --minAnchorSize = 12 --maxDivergence = 30 --concordant --algorithm = blasr --algorithmOptions = --useQuality --maxHits = 1 --hitPolicy = random --seed = 1’ for read alignment, and with ‘variantCaller --skipUnrecognizedContigs haploid -x 5 -q 20 -X120 -v --algorithm = arrow’ for consensus polishing, using PacBio CLR. Variant calls were filtered with merfin version 1.0 to reduce false positives. Two additional rounds of polishing with linked-reads were performed to generate the final polished assembly. In this step, raw-reads were aligned with Longranger align version 2.2.2 and variants were called with Freebayes version 1.3.1^37^ with default parameters. Finally, bcftools version 1.9 (https://github.com/VGP/vgp-assembly/blob/master/dx_applets/bcftools_consensus/asset/Makefile) consensus^38,39^ with options -i 'QUAL > 1 && (GT = "AA" || GT = "Aa")' -Hla was used to generate the consensus.

We generated a complete reference mitochondrial sequence using mitoVGP version 2.2^36^ with standard parameters. The mitogenome was annotated using MITOS2^40^. We merged the mitochondrial assembly with the primary and alternate pseudohaplotypes of the nuclear genome prior to polishing, the mitochondrial genome serving as a sink to avoid overpolishing of the NUMTs. The Etruscan shrew mitogenome was typical of a mammal. It had a total length of 16,982 base pairs and a GC content of 34.74%. We did not detect any issues or anomalies, such as gene duplications.

### Manual curation of the genome assembly

Manual curation of the generated assembly was performed using a previously described protocol by Howe *et al*.^41^. In order to remove contaminants, sequences were screened for trailing ‘N’ bases and clipped and VecScreen revision 87677 (https://www.ncbi.nlm.nih.gov/tools/vecscreen/) was run to remove known adaptors and barcodes. Mitochondrial sequences were removed following a blast check against the assembled mitochondrial genome. Finally the assembly was screened against the RefSeq genomes database for other potential species contamination.

Following contaminant screening, the scaffold assembly was visualized in gEVAL^42^ and the Hi-C contact matrix displayed in HiGlass version 1.11.7^43^ and PretextView version 0.2.3 (https://github.com/wtsi-hpag/PretextView) in order to investigate the assembly and produce a chromosome scale reference. The curation corrected 212 missing or missed joins and removed 28 sequences representing haplotypic duplication. This resulted in a genome with 99.9% of sequence assigned to 22 chromosome-level scaffolds, including X and Y chromosomes.

### Gene ontology (GO) annotation of protein-coding genes

Protein-coding genes were annotated by NCBI (https://www.ncbi.nlm.nih.gov/datasets/gene/GCF_024139225.1/?gene_type=protein-coding). To assign GO terms to protein-coding genes, we used three sequence-based protein function prediction methods: PFP^19^, Phylo-PFP^20^, and ESG^21^. The PFP algorithm uses a scoring method based on E-values to combine GO terms associated with PSI-BLAST^44^ sequence hits, and it then propagates scores to parental terms on the GO directed acyclic graph (DAG) according to the number of known sequences annotated with parent and child terms. Additionally, based on accuracy evaluations over a set of benchmark sequences, it assigns a confidence score to GO term predictions. Phylo-PFP is a modification of PFP that significantly improves the prediction performance by incorporating phylogenetic information in defining sequence similarity. The ESG method performs iterative sequence database searches and annotates a query sequence with GO terms. Each annotation is given a probability based on how similar it is to other sequences in the protein similarity graph.

To capture the significant GO terms annotations, we only considered the predictions with high confidence. The confidence score cutoff for PFP, Phylo-PFP, and ESG is 10,000, 0.7, and 0.4, respectively, and all GO terms with scores above the cutoff are reported in this analysis. To make our predictions more reliable, we also considered the consensus between different prediction methods and reported the GO term predicted as high confidence by any two of the three above-mentioned methods.

### Annotation of protein-coding genes using TOGA

We used TOGA version 1.0 (https://github.com/hillerlab/TOGA)^22^ to assess gene completeness, provide coding gene annotations, and infer orthologs to human and mouse. Briefly, we first computed pairwise genome alignment chains between human (hg38 assembly, GRCh38.p12) and mouse (mm10, GRCm38) as the reference species and the Etruscan shrew as the query species, using lastz version 1.04.15 (parameters K = 2400, L = 3000, Y = 9400, H = 2000, default scoring matrix), axtChain version 1.0 (default parameters except linearGap = loose), RepeatFiller version 1.0, and chainCleaner version 1.0 (default parameters except minBrokenChainScore = 75,000 and -doPairs)^45–47^. We used TOGA version 1.0 with the human GENCODE V38 and mouse GENCODE M25 annotation as input (https://github.com/hillerlab/TOGA/tree/master/TOGAInput). TOGA then infers orthologous gene loci using machine learning and alignments of intronic and intergenic loci, and annotates and classifies orthologous genes. To compare assembly completeness and base accuracy, we considered 18,430 genes that already existed in the placental mammal ancestor^48^ (https://github.com/hillerlab/TOGA/blob/master/TOGAInput/human_hg38/Ancestral_placental.txt) and used a Python script, https://github.com/hillerlab/TOGA/blob/master/supply/TOGA_assemblyStats.py, with the human-referenced TOGA classification to count how many genes have an intact reading frame, inactivating mutations, or missing sequence due to assembly gaps or assembly fragmentation.

### Segmental duplications

**Fig. 2:**
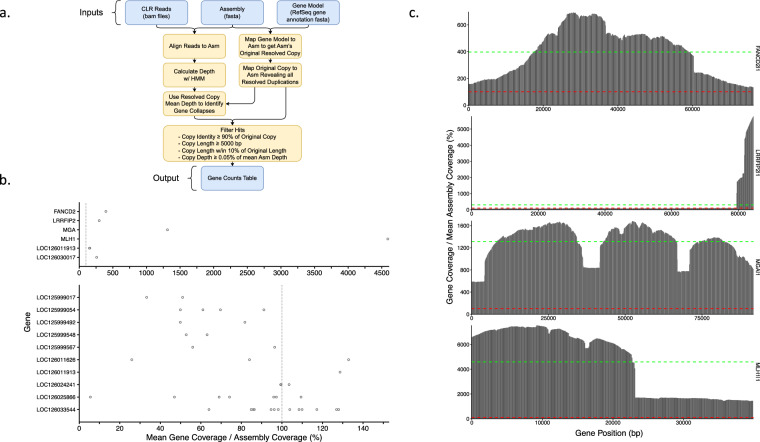
Segmental duplications. (**a**) Segmental Duplication Annotation Pipeline flowchart. (**b**) Mean gene copy depth over assembly depth plotted for all duplicated genes. The top plot highlights genes with collapses. The vertical gray line indicates the mean assembly coverage. (**c**) Coverage maps of partially collapsed genes. Mean coverage over gene and the assembly are shown in green and red respectively.

We identified segmental duplications and the duplicated genes using a combination of self-alignments and read depth (https://github.com/ChaissonLab/SegDupAnnotation2). Our workflow and the overview of duplicated genes are shown in Fig. 2a,b. Briefly, self-alignments enable identification of assembly segments that are highly similar to each other, constituting resolved duplications, while excessive read depth is indicative of collapsed duplications, where two or more copies of a genomic segment had not been resolved by the assembly process. In order to detect collapsed duplications, we mapped CLR reads to the assembly using minimap2 version 2.22 and determined read depth using a hidden Markov model^49,50^. We then used Etruscan shrew RefSeq annotations as gene models to identify duplicated genes also using minimap2 and Needleman Wunsch as implemented by edlib version 1.3.9^51,52^. We were able to identify 15 such genes, six of which had collapsed duplications in the assembly and 10 resolved, with one gene having both (Table 1). Read depths of the four out of six genes with collapsed duplications were inconsistent across the length of the gene, suggesting the presence of truncated copies (Fig. 2c). We annotated such duplications as “partial”. Of the 189,717.5 collapsed kbps detected, 44.2 were in fully collapsed genes and 98,305.9 in partially collapsed genes. There were another 233.3 kbps in resolved duplications. Additionally, we found an 8 kbp segmental duplication to have an insertion in an intronic region of *ADM2*. This duplication was found at 74 loci across 21 chromosomes and is composed of 48% ancient mobile elements (0.66–0.83 similarity to consensus), primarily endogenous retroviruses ERV2-2-I_BT and HERVK, as well as Gypsy elements according to CENSOR^53^. This segmental duplication did not have any BLAST hits in the NCBI *nr/nt* database.

**Table 1:** Estimated copy numbers of duplicated genes.

Gene	Number of resolved copies	Number of collapsed copies	Number of expected copies	Description
MLH1*	1	45	46	mutL homolog 1
MGA*	1	12	13	MAX dimerization protein MGA
LOC126033544	13	0	13	cytochrome c oxidase assembly factor 3 homolog, mitochondrial-like
LOC126025866	7	0	5	NUT family member 2G-like
FANCD2*	1	3	4	FA complementation group D2
LOC126011913	3	2	4	NUT family member 2G-like
LRRFIP2*	1	2	3	LRR binding FLII interacting protein 2
LOC125999054	4	0	3	zinc finger BED domain-containing protein 4-like
LOC126024241	3	0	3	speedy protein E4-like
LOC126030017	1	2	3	zinc finger protein 595-like
LOC125999567	2	0	2	YEATS domain-containing protein 4-like
LOC126011626	3	0	2	arylacetamide deacetylase-like 3
LOC125999017	2	0	1	A-kinase anchor protein 14-like
LOC125999548	2	0	1	YEATS domain-containing protein 4-like
LOC125999492	2	0	1	uncharacterized LOC125999492

The copy count for each gene is shown. The sum of the measured depth over assembly depth is in the ‘Num Expected Copies’ column. Generally, we expect the number of resolved plus collapsed copies to equal expected copies. However, the expected copy count is lower than this sum when read depth per resolved copy of a given gene is sufficiently low compared to the average read depth of the genome (Fig. 2b). This can be caused by some of the resolved gene copies present in the assembly being spurious or, when the duplicated region is heterozygous, by some reads mapping to the alternate haplotype.

*Genes with partial collapsed duplications.

## Data Records

### Genome sequencing and assembly

Raw sequencing and mapping data are available from the VGP GenomeArk repository (https://genomeark.github.io/genomeark-all/Suncus_etruscus.html) and NCBI SRA study SRP456787^54^.

The primary genome assembly was deposited in NCBI GenBank under accession No. GCA_024139225.1^55^. It is also available in Ensembl Rapid Release (https://rapid.ensembl.org/Suncus_etruscus_GCA_024139225.1/Info/Index) and the UCSC Genome Browser (https://genome.ucsc.edu/h/GCF_024139225.1).

The alternate pseudohaplotype was deposited in NCBI GenBank under accession No. GCA_024140225.1^56^. It is also available in the UCSC Genome Browser (https://genome.ucsc.edu/h/GCA_024140225.1).

The mitochondrial genome sequence is available in NCBI GenBank, accession CM044019.1^57^.

### TOGA

TOGA annotations are available from the Senckenberg Genome Browser (https://genome.senckenberg.de/cgi-bin/hgTracks?db=HLsunEtr1) and for download from OSF^58^.

### GO term predictions

GO term predictions are available on OSF^59^. GO Assignments are provided in an Excel file, GO_Prediction_Report_combined.xlsx. It contains the following worksheets:

1. Consensus: the consensus of the predictions from the three methods.
2. ESG: Raw prediction by ESG. Individual scores from ESG are also provided.
3. PhyloPFP: Raw prediction by PhyloPFP. Individual scores from PhyloPFP are also provided.
4. PFP: Raw prediction by PFP. Individual scores from PFP are also provided.

Each worksheet includes information about Gene ID, GO ID, Depth, Class, and GO Description. Here Gene ID is ID of genes, GO ID is the GO term ID, Depth is the depth of the GO ID in the GO DAG, Class is the GO functional category (f- molecular function, p- Biological process, c- Cellular Component), and GO Description describes the GO ID. The result files from PFP, Phylo-PFP, and ESG also include an additional field called Score, which represents the confidence score that the method assigned to that GO term. The Gene Ontology (data-version: releases/2021-11-16) was used for this analysis.

### Segmental duplications

Segmental duplication analysis output is available on OSF^60^.

## Technical Validation

### Assembly quality assessment

Our assembly quality metrics computed with gfastats version 1.3.6^61^ and Merqury version 1.3^29^ are summarized in Table 2. The assembly is partially phased, with 2.5 Gbp primary and 1.5 Gbp alternate pseudohaplotypes. The primary pseudohaplotype is highly contiguous, with scaffold N50 of 132 Mbp and contig N50 of 5 Mbp. The QV of 38 indicates a fairly high base-level accuracy, although somewhat below the VGP target of 40^15^.

**Table 2:** Assembly quality metrics.

Assembly quality metric	Primary pseudohaplotype	Alternate pseudohaplotype
# of scaffolds	148	14,815
Total scaffold length	2,471,683,639	1,515,382,512
Average scaffold length	16,700,565	102,287
Scaffold N50	131,952,996	140,719
Scaffold L50	8	2,910
Scaffold auN	130,872,589	208,812
# of contigs	1,158	14,841
Total contig length	2,461,039,567	1,515,381,692
Average contig length	2,125,250.06	102,107.79
Contig N50	5,042,816	140,198
Contig L50	133	2,912
Contig auN	7,348,256.62	208,676.22
# of gaps in scaffolds	1,010	26
Total gap length in scaffolds	10,644,072	820
Average gap length in scaffolds	10,538.69	31.54
GC content	39.81%	39.87
Merqury QV	37.6497	34.3891
Merqury completeness	95.5922	58.9554

**Fig. 3:**
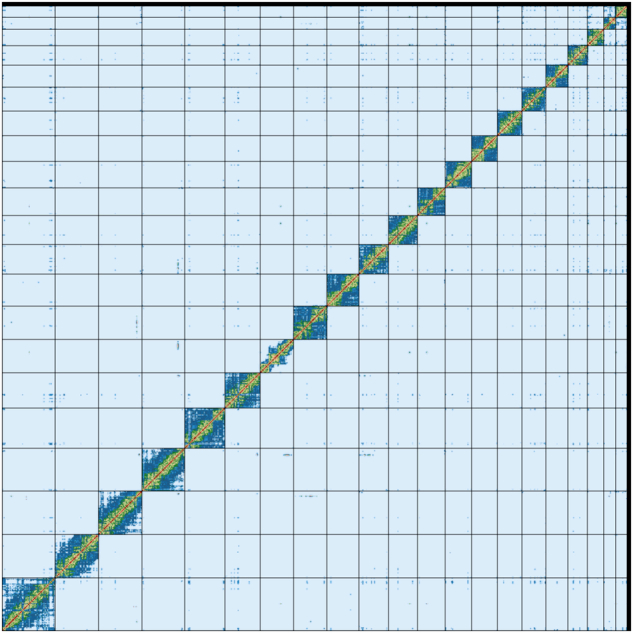
Genome-wide contact map of the curated primary assembly.

The curated primary assembly has been resolved into 20 autosomes and the X and Y sex chromosomes. A genome contact map generated using the PretextMap software (https://github.com/wtsi-hpag/PretextMap) shows that all chromosomes have clean intra-chromosome signals, with minimal inter-chromosome interactions (Fig. 3).

**Fig. 4:**
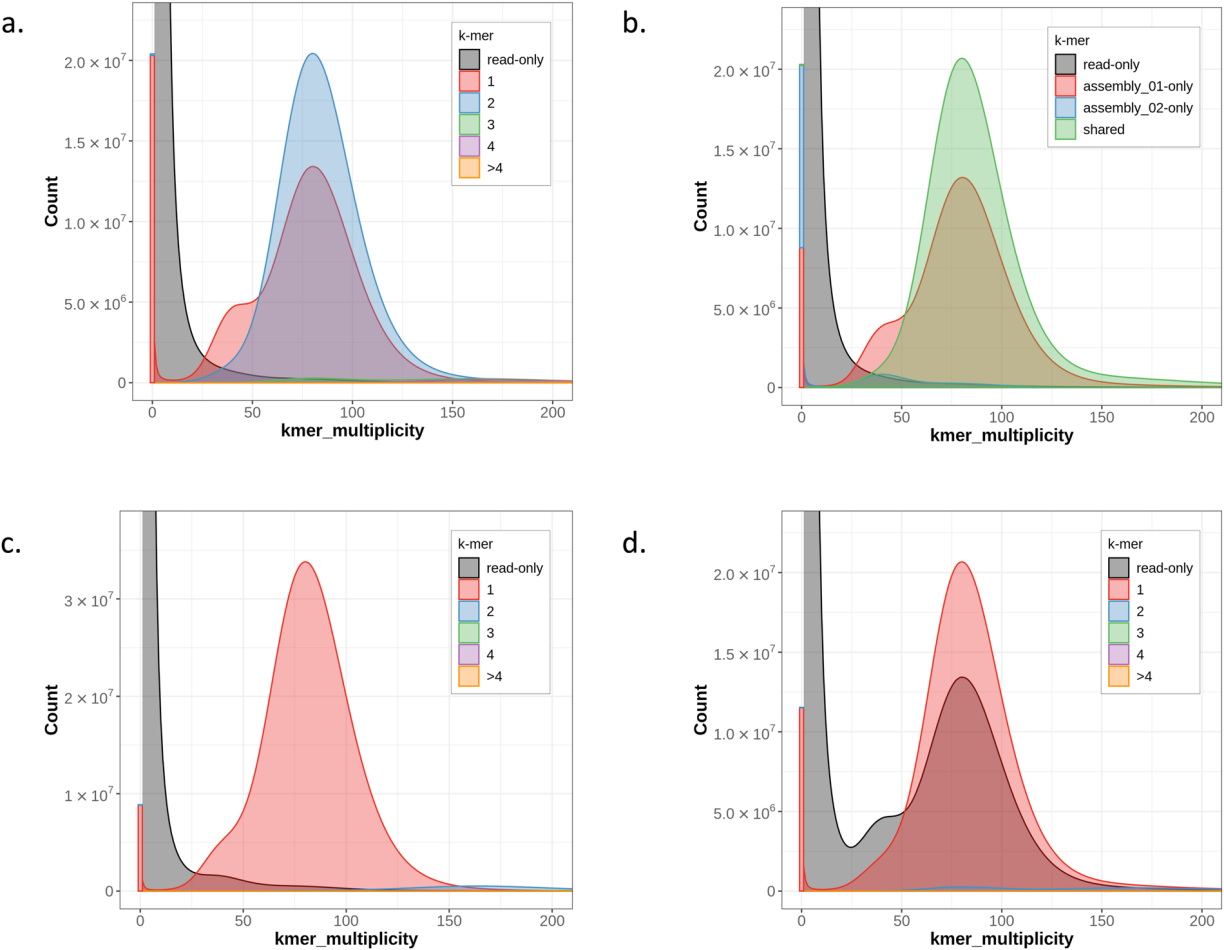
K-mer spectra generated using the Merqury software. (**a**) K-mer spectrum colored by k-mer copy number across the primary and alternate assembly. (**b**) K-mer spectrum colored by which assembly (if any) the k-mer is found in (assembly_01 is the primary, assembly_02 the alternate). (**c**) Primary assembly k-mer spectrum colored by copy number. (**d**) Alternate assembly k-mer spectrum colored by copy number.

*K*-mer spectra for the primary and alternate pseudohaplotypes were computed using the Merqury software version 1.0.0^29^. The spectra are clean, with many diploid regions shared between the two assemblies; however, there are still some homozygous areas missing from the alternate, which is to be expected. The plots do not indicate the presence of false duplicate *k*-mers in the primary assembly (Fig. 4). The primary spectra-cn (Fig. 4c) shows that the primary assembly retains much of the heterozygous regions, but does not have any false duplicates. Accordingly, the alternate spectra-cn (Fig. 4d) has a bump of read-only *k*-mers at haploid coverage, but these are the heterozygous regions that are present in the primary assembly, so they are not actually missing across the two pseudohaplotype assemblies. The primary assembly is the more complete of the two, containing both the homozygous regions as well as heterozygous variants (Fig. 4b).

In addition to high contiguity and sufficient accuracy, the primary assembly is highly complete, having a BUSCO^62,63^ % *Complete* score of 94.9 with *Laurasiatheria* database version 10.

### Comparison with other published genome assemblies within the same mammalian order

**Fig. 5:**
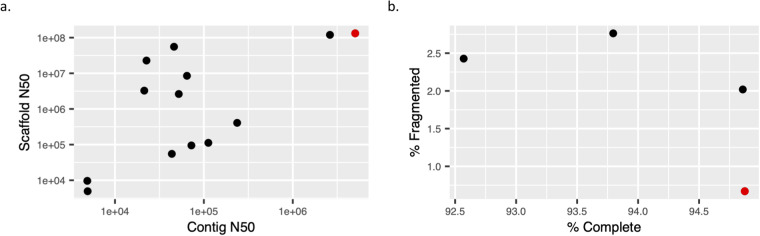
Quality metrics of Eulipotyphla genome assemblies reported by NCBI. The Etruscan shrew assembly is shown in red. (**a**) Contiguity. (**b**) Completeness, as measured by BUSCO scores.

To compare the quality of our genome assembly to other published assemblies of *Eulipotyphla* genomes, we used an R script^64^ to retrieve and plot their quality metrics from the NCBI Assembly database. All of the other assemblies were based on short read technologies, with the exception of *Talpa occidentalis* (Iberian mole)^65^, which also used PacBio CLR, but not the VGP protocols for higher quality phasing, scaffolding, and curation. At the time of writing, our assembly was the most contiguous, having the highest *contig N50* and *scaffold N50* compared to the other assemblies. *Contig N50* values of long-read-based assemblies tend to be orders of magnitude higher than those of short-read-based ones, as evidenced by Fig. 5a. For this study, this translated into having fewer fragmented genes as assessed by BUSCO^62,63^ (Fig. 5b). At the time of writing, BUSCO scores were only available for four *Eulipotyphla* genomes, of which ours had the highest *% Complete* and lowest *% Fragmented* score. The species and genome assembly versions included in this analysis are available on OSF^66^.

We also assessed the status of 18,430 ancestral genes in *Eulipotyphla* genomes that had pre-computed TOGA^22^ results at the time of writing. Our assembly performed about average in terms of the number of intact ancestral open reading frames (ORFs) (Table 3). We had relatively few genes that had missing sequence, reflecting the high contiguity and completeness of our assembly. However, a relatively high number of ancestral genes had inactivating mutations: 2,405, compared to between 940 and 1,483 in other high-quality *Eulipotyphla* genomes. It is likely that many of these apparent mutations are really sequencing errors caused by the lower base-level accuracy of the version of PacBio technology used in this project compared to short read technologies, which could not be fully compensated for by polishing. Despite this issue, our assembly is of sufficiently high quality to serve as a useful reference for transcriptomics and most other purposes. The high contiguity, completeness, and thorough annotation make it a valuable resource for future studies of metabolism and development of one of the world’s smallest mammals.

**Table 3:** TOGA status of 18,430 ancestral placental mammal genes in *Eulipotyphla* genome assemblies.

Assembly	NCBI accession	Species Name	Intact reading frame	Inactivating mutations	Missing sequence
HLgalPyr1	GCA_019455555.1	*Galemys pyrenaicus*	16,783	940	707
HLtalOcc1	GCA_014898055.1	*Talpa occidentalis*	16,711	1,483	236
HLsolPar1	GCA_004363575.1	*Solenodon paradoxus*	16,285	1,131	1,014
HLscaAqu1	GCA_004024925.1	*Scalopus aquaticus*	15,953	1,071	1,406
**HLsunEtr1**	**GCF_024139225.1**	***Suncus etruscus***	**15,288**	**2,405**	**737**
eriEur2	GCF_000296755.1	*Erinaceus europaeus*	14,151	1,131	3,148
conCri1	GCF_000260355.1	*Condylura cristata*	13,913	1,202	3,315
sorAra2	GCF_000181275.1	*Sorex araneus*	12,919	1,331	4,180
HLuroGra1	GCA_004024945.1	*Uropsilus gracilis*	12,584	1,345	4,501
HLcryPar1	GCA_021461705.1	*Cryptotis parvus*	1,423	11,373	5,634

The table is sorted by the number of intact open reading frames. The Etruscan shrew assembly is shown in bold font.

## Data Availability

All code used in this project is publicly available. All relevant software and references are listed in Methods and Technical vation.
